# *Lactiplantibacillus plantarum* improves the growth performance and meat quality of broilers by regulating the cecal microbiota and metabolites

**DOI:** 10.3389/fmicb.2025.1519552

**Published:** 2025-01-28

**Authors:** Yu Liu, Zhisheng Wang, Wenshuo Xi, Junmeng Yuan, Kai Zhang, Huawei Liu, Jinshan Zhao, Yang Wang

**Affiliations:** College of Animal Science and Technology, Qingdao Agricultural University, Qingdao, China

**Keywords:** *Lactiplantibacillus plantarum*, broiler, fatty acids, gut microbiota, gut microbiota metabolites

## Abstract

Gut microbiota can digest and ferment feed into metabolites to influence the meat quality. Probiotics are used to regulate the gut microbiota. In this study, a total of 360 broilers were assigned to 4 treatments (10 broilers per cage): control (Con), low dose of *Lactiplantibacillus plantarum* HW1 (Lp_L), medium dose of Lp (Lp_M) and high dose of Lp (Lp_H) for a 42-day experimental period. Results showed that the Lp treatments improved the growth performance, carcass traits, breast meat quality, and also influenced the fatty acids composition, including the decrease of n-6PUFA/n-3PUFA, and the increase of C18:3n3, ∑n-3PUFA and PUFA/SFA. The lipid metabolism-related gene expressions in the liver showed that Lp treatments increased the expression of *AMPK*, *CPT-1α*, *PPARα*, *ATGL* and also decreased the expression of *PPARγ*, *SREBP-1c*, *ACC*, *FAS*, *LPL*, and *SCD*. Moreover, the abundances of gut microbiota, such as Synergistaceae and *Synergistes* were influenced by the Lp treatments. Functional prediction of the gut microbiota indicated that pathways, including pancreatic secretion and spliceosome were enriched by the Lp treatments. Untargeted metabolomics revealed that the Lp treatments altered the content of metabolites, such as 6-ketomyristic acid and indole-3-acetamide. These metabolites were enriched in pathways including fatty acid metabolism. Correlation analyses revealed potential interactions between growth performance and meat quality, as well as gut microbiota (*Synergistes*, etc.) and metabolites (6-ketomyristic acid, etc.). Overall, our data show that the Lp treatments significantly improved the growth performance, carcass traits and meat quality of broilers by regulating fatty acids, gut microbiota and metabolites.

## 1 Introduction

The meat quality can not only affect consumer purchasing choices, but also influence the health of consumer. It is known that fatty acids, including monounsaturated fatty acids (MUFA), polyunsaturated fatty acids (PUFA), and saturated fatty acids (SFA), are important indicators to evaluate the meat quality, nutritional value and characteristic flavour (Wood et al., 2004). Broiler breast meat contains more PUFA and less SFA than meat from other species of animals, such as beef and lamb (Berzaghi et al., 2005).

The gastrointestinal tract of animals harbors a diverse and complex microbial community. Gut microbiota can digest and ferment food into various metabolites which are utilized by the host to regulate the glucolipid metabolism of skeletal muscle, thereby influencing the meat quality (Liu et al., 2021a). It was reported that the abundance of intestinal unclassified_Erysipelotrichacea*e* and *Butyrivibrio* increased in pigs fed with rice distillers’ by-products and induced an improvement in animal growth and fat deposition (Nguyen Cong et al., 2019). Kang et al. (2022) also suggested that the gut microbiota respond to the wooden breast myopathy by driving dynamic changes in digesta metabolites that eventually enter the plasma.

Probiotics have been shown to promote the growth performance, intestinal health and gut microbiota structure of broilers. Recently, the regulatory effects of probiotics on meat quality have also been reported. Dietary *Bacillus subtilis* supplementation improved meat quality by improving the muscular pH, meat color, water holding capacity, shear force, modifying muscle fiber types and enhancing the antioxidant capacity in broilers (Wang et al., 2024b). *Lactobacillus salivarius* Erya supplementation also improved meat quality in aflatoxin B1-challened broilers, partially by increasing levels of beneficial metabolites, such as SCFA (Chen et al., 2022). Feeding a probiotic mixture containing *Lactobacillus farciminis* and *Lactobacillus rhamnosus* increased the levels of PUFA in the breast meat and decreased SFA level (Eglite et al., 2023). *Lactiplantibacillus plantarum* HW1 (Lp) is a probiotic strain screened from the intestinal mucosa of healthy broilers. In our previous study, we found that Lp can produce various metabolites that have antibacterial ability, including indole lactic acid, paclitaxine A and isocitrate (data not shown). Additionally, dietary Lp supplementation also relieved the necrotic enteritis infection-induced intestinal injury and improved the growth performance in broilers by regulating the gut microbiota (Chen et al., 2023a). However, the role and mechanism of Lp in regulating growth performance and meat quality were unknown. We therefore evaluated different doses of Lp, in terms of the growth performance, carcass trait, meat quality, lipid metabolism, gut microbiota and metabolites.

## 2 Materials and methods

### 2.1 Animal ethical approval

All experimental protocols were approved by the Animal Care and Use Committee of Qingdao Agricultural University (protocol number 20230720351). We have followed the ARRIVE guidelines for reporting animal research.

### 2.2 Lp culture and preparation

Lp was isolated from intestinal mucosa of healthy broilers and deposited in the China General Microbiological Culture Collection Center (Beijing, China; CGMCC No. 26160). Cryopreserved Lp was activated and cultured in MRS medium (Haibo Biotechnology Co., Ltd., Qingdao, China) at 37°C for 24 h. Thereafter, the liquid solution of Lp was diluted until the concentration of bacteria reached the required concentration and then centrifuged at 3000 × g for 10 min to obtain the Lp pellet, and diluted in drinking water.

### 2.3 Experimental design

A total of 360 one-day old male AA broilers with similar initial body weights (42 ± 0.13 g) were assigned to 4 treatments with 9 replicates per treatment (10 broilers per cage). The 4 treatments were: control (Con), low dose of Lp (Lp_L), medium dose of Lp (Lp_M) and high dose of Lp (Lp_H). Broilers in the Con treatment drank normal water. Broilers in the Lp_L, Lp_M and Lp_H treatments firstly consumed 200 mL water containing 5 × 10^5^ CFU/mL, 5 × 10^6^ CFU/mL and 5 × 10^7^ CFU/mL Lp, respectively, and then were allowed free access to normal drinking water according to previous published methods (Lokapirnasari et al., 2017; Atela et al., 2019). All the broilers were kept in cages of 100 cm × 100 cm × 60 cm (length × width × high), and lighting conditions were managed 23 L:1 D throughout the study. The temperature of the room was set at 33–35°C during the first week, and then decreased by 2°C every week to 24°C. Broilers in all groups were fed a basal diet, which was prepared according to the requirements of the National Research Council (NRC, 1994; Table 1). The experimental period was 42 days.

**Table 1:** Ingredients and nutrient levels in the basal diet %.

Item	Contents	
	1–21 days of age	22–42 days of age
Ingredients (%, as fed)		
Corn	57.00	62.00
Soybean meal	36.50	30.00
Soybean oil	3.90	5.60
Limestone	1.60	1.40
CaHPO_4_	0.40	0.35
NaCl	0.25	0.30
DL-Methionine	0.15	0.15
Premix^1^	0.20	0.20
Total	100	100
Total nutrient levels^2^ (%, as fed)		
Crude protein	20.98	18.53
Calcium	0.90	0.80
Available phosphorus	0.47	0.42
Lysine	1.18	1.03
Methionine	0.45	0.42
Metabolizable energy (MJ/kg)	12.77	13.39

^1^Provided per kilogram of diet: Fe (as ferrous sulfate) 80 mg; Cu (as copper sulfate) 10 mg; Zn (as zinc sulfate) 75 mg; Mn (as manganese sulfate) 80 mg; Se (as sodium selenite) 0.30 mg; I (as potassium iodide) 0.40 mg. Vitamin A (trans-retinyl acetate) 8,000 IU; Vitamin D3 (cholecalciferol) 3,000 IU; Vitamin E (all-rac-α-tocopherol acetate) 20 IU; Vitamin K3 (menadione) 2.0 mg; Vitamin B1 (thiamin) 4.2 mg; Vitamin B2 (riboflavin) 4.0 mg; Vitamin B6 (pyridoxine HCl) 4.5 mg; Vitamin B12 (cobalamin) 0.02 mg; nicotinic acid 10 mg; calcium pantothenate 11 mg; folic acid 1.0 mg; biotin 0.15 mg.

^2The nutrient levels were calculated values.^

### 2.4 Sample collection

On day 42, after 12 h fasting, one broiler from each replicate was randomly selected. Blood samples were collected from the wing vein into vacuum tubes with coagulant and centrifuged at 3000 × *g* at 4°C for 10 min, the separated serum samples were placed in a 1.5 mL Eppendorf tubes and stored at −20°C for further use. The spleen, thymus, bursa of Fabricius, liver and breast muscle were collected for carcass traits analyses. A portion of the left breast muscle was temporarily stored at 4°C for meat quality analyses. Samples of the remaining breast muscle, liver and cecal contents were placed in liquid nitrogen immediately and then stored at −80°C for further use.

### 2.5 Analyses of growth performance and carcass traits

The amounts of provided and refused feed were measured weekly on a replicate basis to calculate the average daily feed intake (ADFI). Body weight (BW) was measured at d 21 and d 42 to calculate average daily gain (ADG), and feed:gain ratio (F:G) on a per replicate basis.

On day 42, according to the method by Zhu et al. (2022), one broiler from each replicate was killed by bloodletting of jugular vein and were manually dissected to dressed, half-eviscerated, eviscerated, breast muscle and thigh muscle weight and yield. Dressed rate (%) = 100 × dressed weigh/BW; half-eviscerated rate (%) = 100 × half-eviscerated weight/BW; eviscerated rate (%) = 100 × eviscerated weight/BW; breast muscle rate (%) = 100 × breast muscle weight/eviscerated weight; thigh muscle rate (%) = 100 × thigh muscle weight/eviscerated weight.

### 2.6 Meat quality analysis

The meat color parameters, including lightness (L*), redness (a*) and yellowness (b*), were evaluated using a colorimeter (CR-410, Konica Minota, Tokyo, Japan) at 45 min and 24 h after slaughter (Chen et al., 2017). The meat pH value at 45 min (pH_45min_) and 24 h (pH_24h_) after slaughter was detected using a pH meter (pH-Seven2Go, Mettler Toledo, Germany) equipped with a glass electrode and metal thermometer probe inserted 1 cm into the superior portion of the breast muscle (Liu et al., 2021b). The drip loss of the breast muscle was measured according to Wang et al. (2017), the breast muscles trimmed into strips (1 × 1 × 2 cm) were weighed (M1), covered with plastic bags and hung in a refrigerator at 4°C for 24 h with thin lines parallel to the direction of muscle fibers. After removing the plastic bags and thin lines, filter papers were used to absorb the surface moisture and then weigh the samples again (M2). Drip loss (%) = [(M1 – M2)/M1] × 100%. The cooking loss of the breast muscle was measured according to Cong et al. (2017), the breast muscles were weighed (W1) and covered with polyethylene bags, cooked in a water bath at 80°C for 15 min to reach an internal temperature of 75°C. Then, the cooked samples were cooled to room temperature, wiped with absorbent paper and reweighed (W2). Cooking loss (%) = [(W1 – W2)/W2] × 100%. The shear force following the method described by Wang et al. (2024a) was measured using a digital meat tenderness meter (model C-LM3, Shandong, China), the breast muscles along the direction of the myofibrils were cut into three strips (1 × 1 × 4 cm). Each strip was measured and then averaged and recorded in Newtons. Intramuscular fat (IMF) content was determined using Soxhlet extraction method with diethyl ether anhydrous as the solvent, in accordance with the standard method “Determination of Fat in Food” from the National Standard for Food Safety (GB/T 5009.6–2016).”

### 2.7 Real-time quantitative PCR (RT-qPCR)

Refer to the method of our previous research to extract total RNA (Liu et al., 2022a). Total RNA was isolated from the liver samples using the Trizol reagent (Tiangen Biochemical Technology Co., Ltd., Beijing, China), and the purity and concentration of RNA were detected using a spectrophotometer (NanoDrop 2000c, Thermo Fisher Scientific, Waltham, MA) and agarose gel electrophoresis. cDNA was prepared using a TB Green Premix Ex Taq kit (TaKaRa). The mRNA expressions of adenosine monophosphate activated protein kinase (*AMPK*), peroxisome proliferator-activated receptor alpha (*PPARα*), peroxisome proliferator-activated receptor gamma (*PPARγ*), sterol regulatory element binding protein-1c (*SREBP-1c*), acetyl-CoA carboxylase (*ACC*), fatty acid synthase (*FAS*), adipose triglyceride lipase (*ATGL*), lipoprotein lipase (*LPL*), carnitine palmityl transferase (*CPT-1α*) and stearoyl-CoA desaturase (*SCD*) were determined by RT-qPCR using a BioRad CFX96 Real-Time PCR system (Bio-Rad Laboratories, Hercules, CA). The thermocycle protocol was: 95°C for 30 s, 95°C for 5 s with 40 cycles, and 60°C for 34 s. Then, the purity of the PCR product was monitored by a melting curve. The 2^−ΔΔCq^ method was used to calculate target gene relative expressions. *β*-actin was used as the reference gene. Primer 5.0 and oligo 7.0 software were used for the PCR primer sequence design (Supplementary Table S1).

### 2.8 Fatty acids analysis

After weighed and homogenized, 300 μL of breast meat homogenate supernatant was collected by centrifugation. Then, 15 μL 500 mg/L methyl salicylate (Sigma-Aldrich, Shanghai, China) was added into the supernatant as an internal standard. The mixture was vortexed to GC–MS analysis (Hoving et al., 2018). Mixed standard of 51 fatty acid methyl esters was obtained from NU-CHEK-PREP (Shanghai, China).

The GC analysis was performed by Panomix Biomedical Tech Co., Ltd. (Suzhou, China) on trace 1,300 gas chromatograph (Thermo Fisher Scientific, USA). The GC was fitted with a capillary column Thermo TG-FAME (50 m*0.25 mm ID*0.20 μm) and helium was used as the carrier gas at 0.63 mL/min. Injection was made in split mode at 8:1 with an injection volume of 1 μL and an injector temperature of 250°C. The temperature of the ion source and transfer line were 300°C and 280°C, respectively. The column temperature was programmed to increase from an initial temperature of 80°C, which was maintained for 1 min, followed by an increase to 160°C at 20°C/min, which was maintained for 1.5 min, and increase to 196°C at 3°C/min, which was maintained for 8.5 min, and finally to 250°C at 20°C/min and kept at this temperature for 3 min. Mass spectrometric detection of metabolites was performed on ISQ 7000 (Thermo Fisher Scientific, USA) with electron impact ionization mode. Single ion monitoring (SIM) mode was used with the electron energy of 70 Ev (Beccaria et al., 2018).

### 2.9 DNA extraction and microbiomic analysis

Samples of cecal content (0.1 g) were sent to Metware Biotechnology Co., Ltd. (Wuhan, China) for 16S rRNA analysis using the Illumia MiSeq platform. Total genome DNA from samples was extracted using CTAB method. DNA quality was confirmed using 1% agarose gel electrophoresis. DNA samples were amplified by PCR using bar-coded primers flanking the V3-V4 region of the 16S rRNA genes. The V3-V4 hypervariable region of the 16S rRNA gene was amplified using the primer pair 341F (5’-CCTAYGGGRBG CASCAG-3′) and 806R (5′- GGACTACNNGGGTATCTAAT-3′). The thermal cycling consisted of initial denaturation for 1 min at 98°C, 30 cycles of 10 s at 98°C, annealing for 30 s at 50°C, elongation for 30 s at 72°C, and finally for 5 min at 72°C. High-quality clean tags were generated by quality filtering of the raw tags based on the fastp (v0.22.0, https://github.com/OpenGene/fastp). Operational taxonomic units (OTUs) were clustered at 97% sequence similarity using Uparse software (USEARCH v7, http://www.drive5.com/uparse/) (Edgar, 2013), and representative sequences of each cluster were used to assign taxonomy through annotation against the SILVA database (http://www.arb-silva.de/) (Quast et al., 2013). Indices of alpha diversity of the samples were evaluated. Principal coordinate analysis (PCoA) was undertaken for all samples following analysis through application of Braye-Curtis dissimilarity and unweighted UniFrac using R phyloseq (v1.40.0).

Taxonomic classification was achieved based on homology (>97% identity) between queried and reference sequences from the SILVA138.1 (http://www.arb-silva.de/) SSUrRNA database (Quast et al., 2013). Subsequently, the OTU table was normalized using MAFFT (v7.520, https://mafft.cbrc.jp/alignment/software/), followed by metagenome functional prediction based on the Kyoto Encyclopedia of Genes and Genomes (KEGG) database. Significant differences in gene function among the groups were revealed by the Kruskale-Wallis test. All DNA datasets were submitted to the NCBI Sequence Read Archive database under BioProject ID PRJNA1077732.

### 2.10 Metabolomic analysis

The untargeted metabolomic analyses were performed by Metware Biotechnology Co., Ltd. (Wuhan, China). Samples of the cecal content were lyophilized, ground, and crushed on ice. The thawed samples were homogenized by a grinder at 30 HZ for 20 s. A 400 μL solution (methanol:water = 7:3, V/V) containing internal standard was added in to 20 mg ground sample, and shaked at 1500 × *g* for 5 min. After placing on ice for 15 min, the sample was centrifuged at 12000 × *g* for 10 min at 4°C. A 300 μL of supernatant was collected and placed in −20°C for 30 min. The sample was then centrifuged at 12000 × *g* for 3 min at 4°C. A 200 μL aliquots of supernatant were transferred for LC–MS analysis.

All samples were for two LC/MS methods. One aliquot was analyzed using positive ion conditions and was eluted from T3 column (Waters ACQUITY Premier HSS T3 Column 1.8 μm, 2.1 mm * 100 mm). The mobile phase consisted of 0.1% formic acid in water (solvent A) and acetonitrile (solvent B). The analytical conditions were: column temperature, 40°C; flow rate, 0.4 mL/min; injection volume, 4 μL; Another aliquot was using negative ion conditions. The data acquisition was operated using Analyst TF 1.7.1 Software (Sciex, Concord, ON, Canada). The laboratory’s self-built database, integrated public database, AI database and metDNA were used for substance identification. The “SVR” method was used to correct the peak area. The peaks with detetion rate lower than 50% in each group of samples were discarded. Orthogonal partial least squares discriminant analysis (OPLS-DA) was performed using R package MetaboAnalystR. The differential metabolites were determined by VIP (VIP > 1) and *p*-value (p-value <0.05, Student’s t test). Screening criteria: *p* < 0.01, metabolites with fold-change (FC) ≥ 2 and ≤ 0.5. The KEGG Pathway database (http://www.kegg.jp/kegg/pathway.Html) was used to annotate the differential metabolites. The specific metabolomic data analysis referred to our previous research (Zhao et al., 2023).

### 2.11 Statistics

Growth performance, carcass traits, breast meat quality and genes expressions were assessed by one-way ANOVA using SPSS 20.0. When effects were significant (*p* < 0.05), Tukey’s multiple range test was used to identify significant differences among the groups. The tabulated results are shown as means and SEM derived from the ANOVA error mean square. Spearman’s rank correlation analysis was performed to determine the relationships between the growth performance, meat quality and cecal bacteria as well as metabolites. Clustering Spearman’s correlation heatmap with signs was performed using the Metware Cloud at https://cloud.metware.cn. All means and comparison groups were considered statistically significant at *p* < 0.05.

## 3 Results

### 3.1 Growth performance and carcass traits of broilers

Table 2 showed that all the Lp treatments led to higher final BW (*p* < 0.01), and broilers in the Lp_H group had the highest BW (*p* < 0.01). During d 22 to d 42, the Lp_M and Lp_H treatments increased the ADG compared with the Con and Lp_L groups (*p* < 0.01). During d 1 to d 42, all the Lp treatments elevated the ADG in comparison with the Con group (*p* < 0.01). Broilers in the Lp_H group had the highest ADG during d 22 to d 42 and d 1 to d 42 compared with the other groups (*p* < 0.01). As for the F:G, during d 22 to d 42, high level Lp supplementation decreased the F:G compared with the Con group (*p* = 0.04).

**Table 2:** Effects of Lp on the growth performance of broilers.

Items	Days	Con	Lp_L	Lp_M	Lp_H	SEM	p-Value
BW (g)	0	42.28	42.17	42.11	42.17	0.13	0.64
	21	814.14	816.70	818.09	818.23	1.69	0.08
	42	2470.51^c^	2505.12^b^	2512.18^b^	2550.02^a^	13.77	<0.01
ADG (g)	1–21	36.76	36.88	36.95	36.96	0.08	0.08
	22–42	78.87^c^	80.40^bc^	80.67^b^	82.47^a^	0.65	<0.01
	1–42	57.82^c^	58.64^b^	58.81^b^	59.71^a^	0.33	<0.01
ADFI (g)	1–21	45.23	45.33	45.32	45.93	0.26	0.04
	22–42	147.24	147.35	147.58	147.68	2.04	0.99
	1–42	96.24	96.34	96.45	96.81	1.00	0.95
F:G	1–21	1.23	1.23	1.23	1.24	0.01	0.10
	22–42	1.87^a^	1.83^ab^	1.83^ab^	1.79^b^	0.03	0.04
	1–42	1.66	1.64	1.64	1.62	0.02	0.12

^a,b,c^ Mean values within a row with no common superscript differ significantly (*P* < 0.05). SEM, standard error of the mean (*n* = 9).

Table 3 suggested that all the Lp treatments increased the percentage of eviscerated yield and breast muscle rate compared with the Con group (*p* < 0.01). Moreover, compared with the Lp_L and Lp_M groups, Lp_H induced a higher breast muscle rate (*p* < 0.01).

**Table 3:** Effects of Lp on the carcass traits of broilers.

Traits	Con	Lp_L	Lp_M	Lp_H	SEM	p-Value
Dressed rate (%)	93.20	93.32	93.13	93.27	0.12	0.37
Half-eviscerated rate (%)	88.11	87.48	88.11	88.22	0.36	0.18
Eviscerated rate (%)	69.81^b^	71.05^a^	71.11^a^	71.32^a^	0.34	<0.01
Breast muscle rate (%)	29.27^c^	33.63^b^	34.89^b^	40.89^a^	0.54	<0.01
Thigh muscle rate (%)	22.35	22.53	22.35	23.08	0.30	0.06

^a,b,c^ Mean values within a row with no common superscript differ significantly (*p* < 0.05). SEM, standard error of the mean (*n* = 9).

### 3.2 Breast meat quality of broilers

The pH_24h_ (*p* = 0.03), a*_45min_ (*p* < 0.01) and IMF (*p* = 0.04) of breast meat was increased by the Lp_H treatment. The L*_24h_ (*p* = 0.04) of breast meat was decreased by the Lp_H treatment. Moreover, all the Lp treatments decreased the b*_45min_. In addition, the Lp_M and Lp_H treatments elevated the a*_24h_ compared with the Con and Lp_L groups (*p* < 0.01) (Table 4).

**Table 4:** Effects of Lp on the breast meat quality of broilers.

Items	Con	Lp_L	Lp_M	Lp_H	SEM	p-Value
Shear force(N)	13.30	12.85	13.07	12.69	0.23	0.07
Drip loss (%)	2.99	2.61	2.83	2.75	0.16	0.15
Cooking loss (%)	25.45	24.17	23.88	22.87	0.97	0.09
IMF (%)	2.91^b^	2.92^b^	3.06^ab^	3.36^a^	0.16	0.04
pH_45min_	6.56	6.47	6.44	6.51	0.07	0.44
pH_24h_	5.91^b^	6.12^ab^	6.12^ab^	6.19^a^	0.09	0.03
L*_45min_	42.98	43.19	42.10	42.14	0.48	0.06
a*_45min_	7.20^b^	7.12^b^	7.48^ab^	7.60^a^	0.14	0.01
b*_45min_	16.52^a^	13.93^b^	13.17^bc^	12.97^c^	0.32	<0.01
L*_24h_	46.45^a^	45.38^ab^	44.47^ab^	43.95^b^	0.87	0.04
a*_24h_	6.63^b^	6.58^b^	7.02^a^	7.16^a^	0.16	<0.01
b*_24h_	14.79	14.94	14.51	14.07	0.41	0.18

L*, lightness; a*, redness; b*, yellowness; IMF, intramuscular fat.

^a,b,c^ Mean values within a row with no common superscript differ significantly (*P* < 0.05). SEM, standard error of the mean (*n* = 9).

### 3.3 Deposition of fatty acids in breast meat of broilers

All the Lp treatments decreased the n-6PUFA/n-3PUFA (*p* = 0.01), and increased the levels of C18:3n3, C22:6n3 and ∑n-3PUFA (*p* < 0.01). The Lp_H treatment decreased the C16:0 level and ∑SFA (*p* < 0.01) and increased the PUFA/SFA (*p* < 0.01) compared with the other groups (*p* < 0.01), decreased the level of C18:0 compared with the Con and Lp_L group (*p* = 0.02), increased the C20:5n3 level compared with the Con group (*p* < 0.01) and increased the C22:5n6 level compared with the Con and Lp_M groups (*p* < 0.01) (Table 5).

**Table 5:** Effects of Lp on the deposition of fatty acids in breast meat of broilers.

Items	Con	Lp_L	Lp_M	Lp_H	SEM	p-Value
C14:0	6.92	6.12	6.34	6.47	0.83	0.80
C16:0	576.91^a^	544.97^a^	578.96^a^	380.86^b^	57.53	<0.01
C18:0	221.53^a^	220.89^a^	209.59^ab^	168.07^b^	17.84	0.02
C18:2n6	435.12	471.53	484.02	486.30	31.09	0.35
C18:3n3	17.69^b^	23.42^a^	22.66^a^	24.85^a^	1.28	<0.01
C20:0	1.14	1.39	1.28	1.35	0.18	0.53
C20:3n6	22.67	24.66	23.50	23.83	1.47	0.61
C20:4n6	95.51	97.86	93.97	101.71	6.23	0.63
C20:5n3	4.14^b^	4.85^ab^	4.95^ab^	5.92^a^	0.42	<0.01
C22:6n3	6.20^b^	8.66^a^	8.60^a^	9.16^a^	0.54	<0.01
∑SFA	813.53^a^	780.15^a^	802.82^a^	564.00^b^	70.45	<0.01
∑MUFA	616.30	629.73	605.78	669.72	49.46	0.60
∑PUFA	655.21	708.17	713.28	733.04	32.74	0.14
∑n-6PUFA	612.51	653.86	660.07	674.93	32.52	0.29
∑n-3PUFA	42.70^b^	54.31^a^	53.21^a^	58.11^a^	1.87	<0.01
PUFA/SFA	0.81^b^	0.94^b^	0.92^b^	1.31^a^	0.10	<0.01
n-6PUFA/n-3PUFA	14.34^a^	12.14^b^	12.41^b^	11.62^b^	0.78	0.01

Explanation of abbreviations: ∑SFA: saturated fatty acid; ∑MUFA: monounsaturated fatty acid; ∑PUFA: polyunsaturated fatty acid; ∑n-6PUFA: total omega 6 fatty acids; ∑n-3PUFA: total omega 3 fatty acids.

^a,b,c^ Mean values within a row with no common superscript differ significantly (*P* < 0.05). SEM, standard error of the mean (*n* = 6).

### 3.4 Expressions of lipid metabolism-related genes in the liver

All the Lp treatments increased the expression of *AMPK* and *CPT-1α* (*p* < 0.01), and also decreased the expression of *PPARγ*, *SREBP-1c*, *LPL* and *SCD* (*p* < 0.01). The Lp_M and Lp_H treatments increased the *ATGL* expression compared with the other two groups and decreased the *ACC* expression compared with the Con group. The Lp_H treatment increased the *PPARα* (*p* = 0.03) compared with the Con group and decreased the *FAS* expressions (*p* < 0.01) compared with the Con group (Table 6).

**Table 6:** Effects of Lp on the expressions of lipid metabolism-related genes in the liver of broilers.

Genes	Con	Lp_L	Lp_M	Lp_H	SEM	p-Value
*AMPK*	1.00^b^	1.60^a^	1.67^a^	2.00^a^	0.24	<0.01
*PPARα*	1.00^b^	1.52^ab^	1.69^ab^	1.85^a^	0.28	0.03
*PPARγ*	1.00^a^	0.61^b^	0.58^b^	0.34^b^	0.13	<0.01
*SREBP-1c*	1.00^a^	0.48^b^	0.35^c^	0.23^c^	0.06	<0.01
*ACC*	1.00^a^	0.69^ab^	0.58^b^	0.45^b^	0.14	<0.01
*FAS*	1.00^a^	0.70^ab^	0.61^ab^	0.53^b^	0.17	0.04
*ATGL*	1.00^b^	1.11^b^	1.56^a^	1.60^a^	0.13	<0.01
*LPL*	1.00^a^	0.50^b^	0.46^b^	0.42^b^	0.21	0.02
*CPT-1α*	1.00^b^	1.88^a^	1.81^a^	2.24^a^	0.21	<0.01
*SCD*	1.00^a^	0.55^b^	0.47^b^	0.37^b^	0.14	<0.01

AMPK, adenosine monophosphate activated protein kinase; PPARα, peroxisome proliferator-activated receptor alpha; PPARγ, peroxisome proliferator-activated receptor gamma; SREBP-1c, sterol regulatory element binding protein-1c; ACC, acetyl-CoA carboxylase; FAS, fatty acid synthase; ATGL, adipose triglyceride lipase; LPL, lipoprotein lipase; CPT-1α, carnitine palmityl transferase; SCD, stearoyl-CoA desaturase.

^a,b,c^ Mean values within a row with no common superscript differ significantly (*P* < 0.05). SEM, standard error of the mean (*n* = 6).

### 3.5 Cecal microbiota

**Figure 1:**
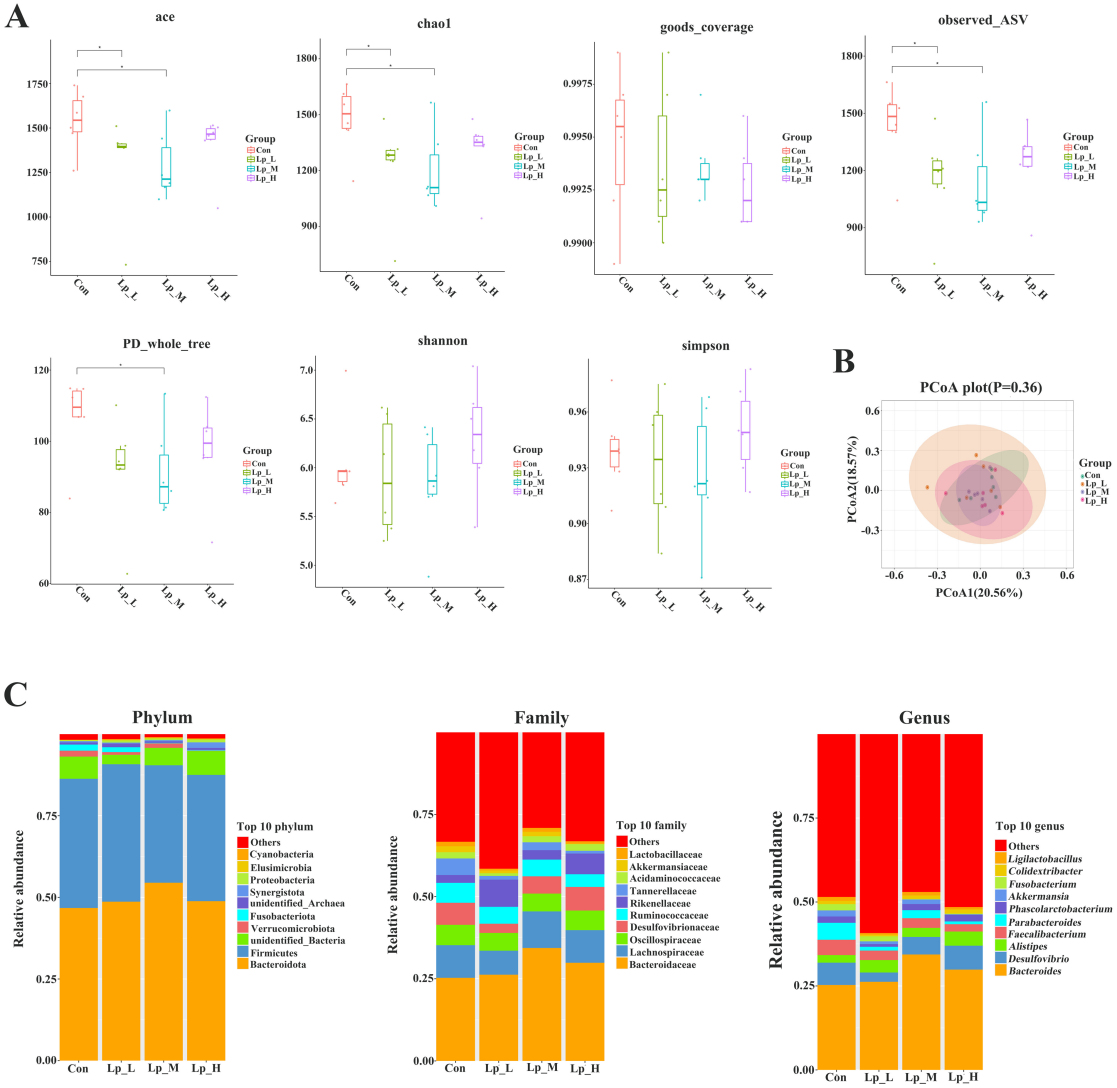
Effects of Lp on the *α*-diversity **(A)**, *β*-diversity **(B)** and abundance **(C)** of cecal microbiota of broilers. *n* = 6.

As for the bacterial diversity, the Lp_L and Lp_M treatments decreased the values of ace, chao1, and observed_ASV (*p* < 0.05). The Lp_M treatment also reduced the PD_whole_tree value (*p* < 0.05) (Figures 1A,B).

Compared with the Con group, the Lp_L treatment decreased the abundance of bacteria including Muribaculaceae and Tannerellaceae at family level (*p* < 0.05), and *Merdibacter*, *Colidextribacter*, *Parabacteroides* at genus level (*p* < 0.05); the Lp_M treatment decreased the abundance of Muribaculaceae at family level (*p* < 0.05); the Lp_H treatment increased the abundance of Synergistaceae at family level and *Synergistes* at genus level (*p* < 0.05), and decreased the abundance of Tannerellaceae at family level and *Parabacteroides* at genus level (*p* < 0.05) (Figure 1C and Supplementary Tables S2–S4).

**Figure 2:**
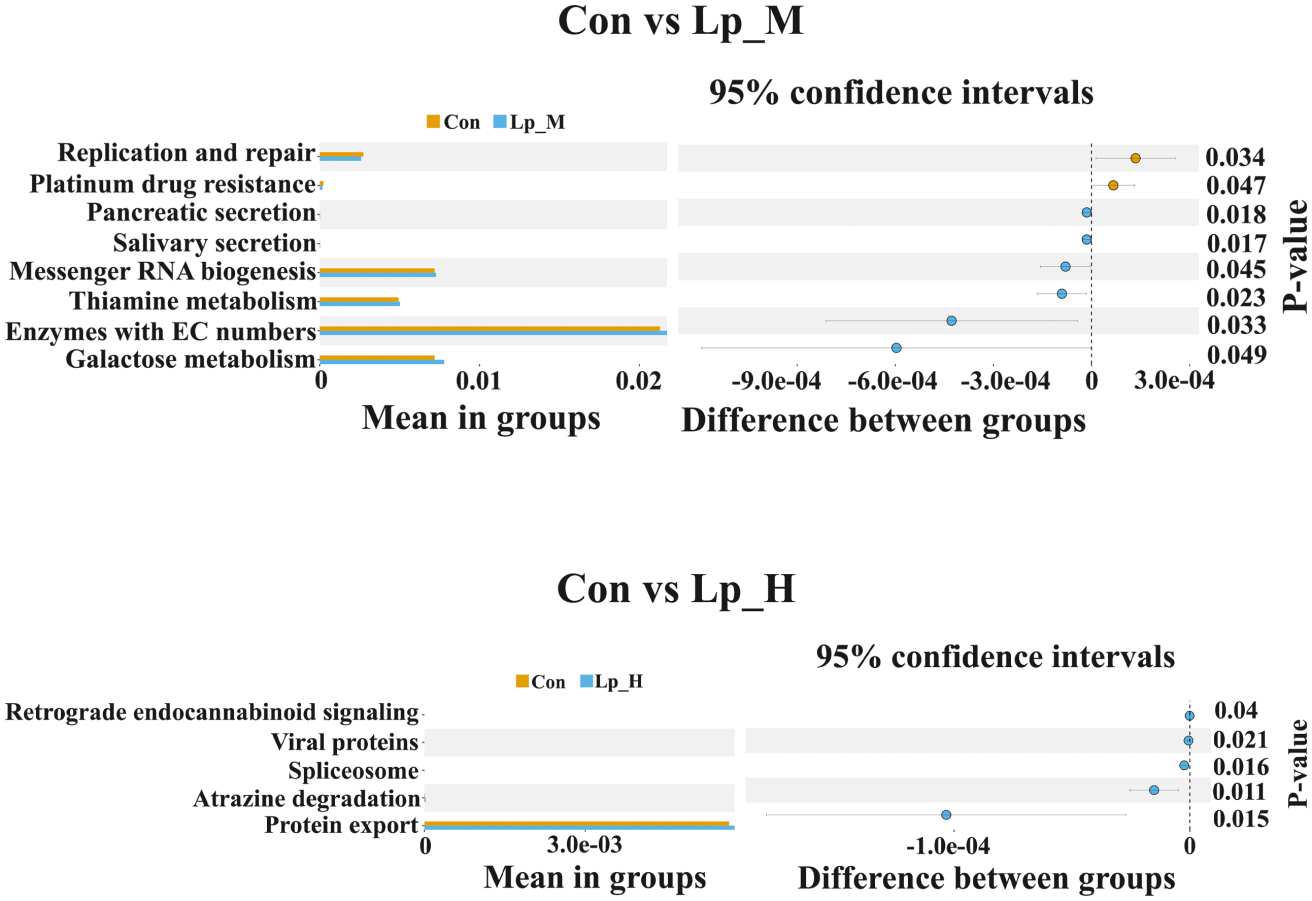
Effects of Lp on the functional prediction of microflora of broilers. *n* = 6.

PICRUSt analysis indicated that compared with the Con group, the pathways including replication and repair, and plantinum drug resistance were less enriched in the Lp_M group (*p* < 0.05). Pathways such as pancreatic secretion, salivary secretion, messenger RNA biogenesis, thiamine metabolism, enzymes with EC numbers and galactose metabolism were enriched in the Lp_M group (*p* < 0.05). Additionally, the pathways including retrograde endocannabinoid signaling, viral proteins, spliceosome, atrazine degradation and protein export (*p* < 0.05) (Figure 2).

### 3.6 Cecal microbiota metabolites

**Figure 3:**
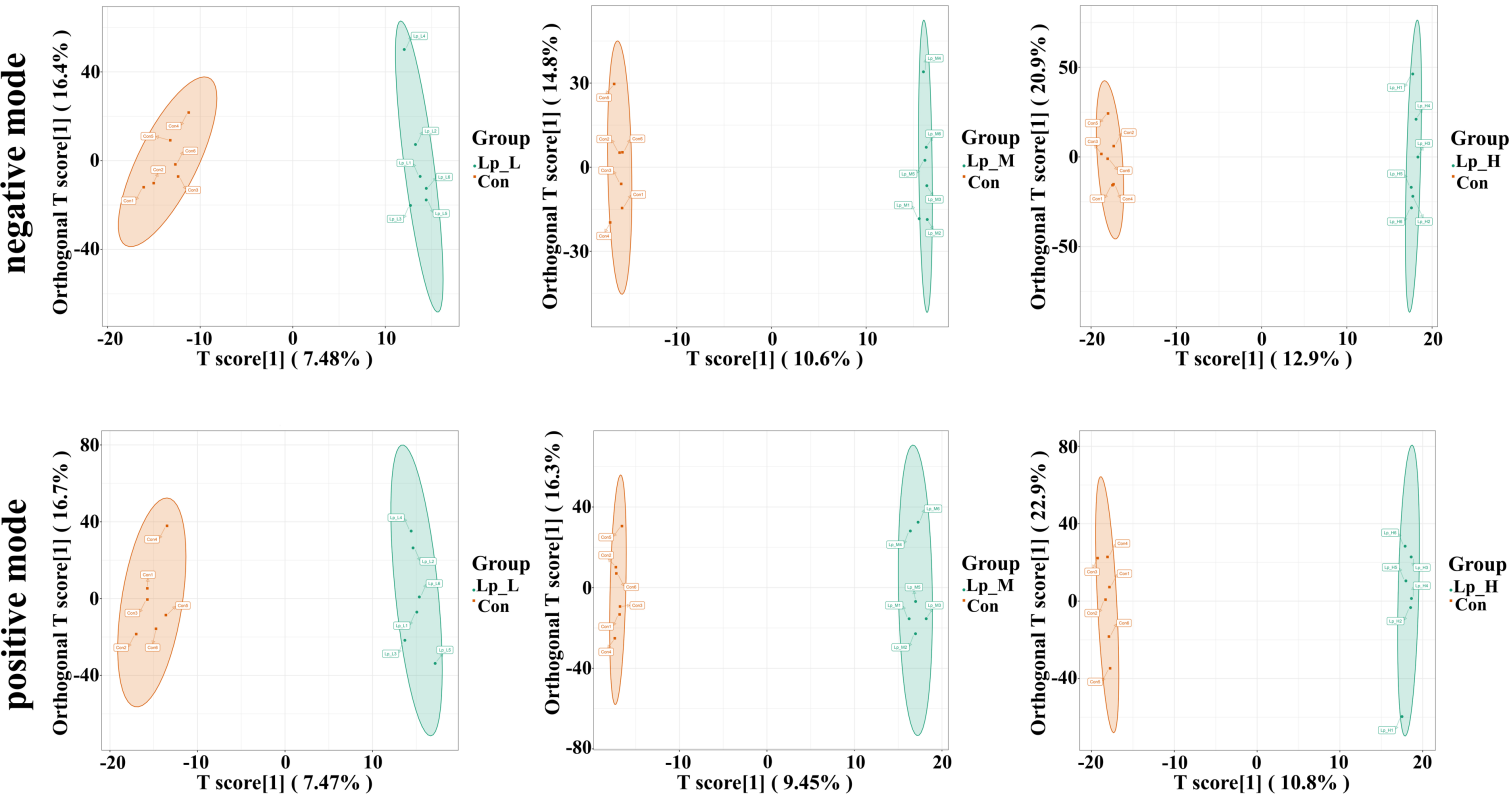
Effects of Lp on the metabolites of the gut microbiota of broilers by OPLS-DA scores plot. *n* = 6.

**Figure 4:**
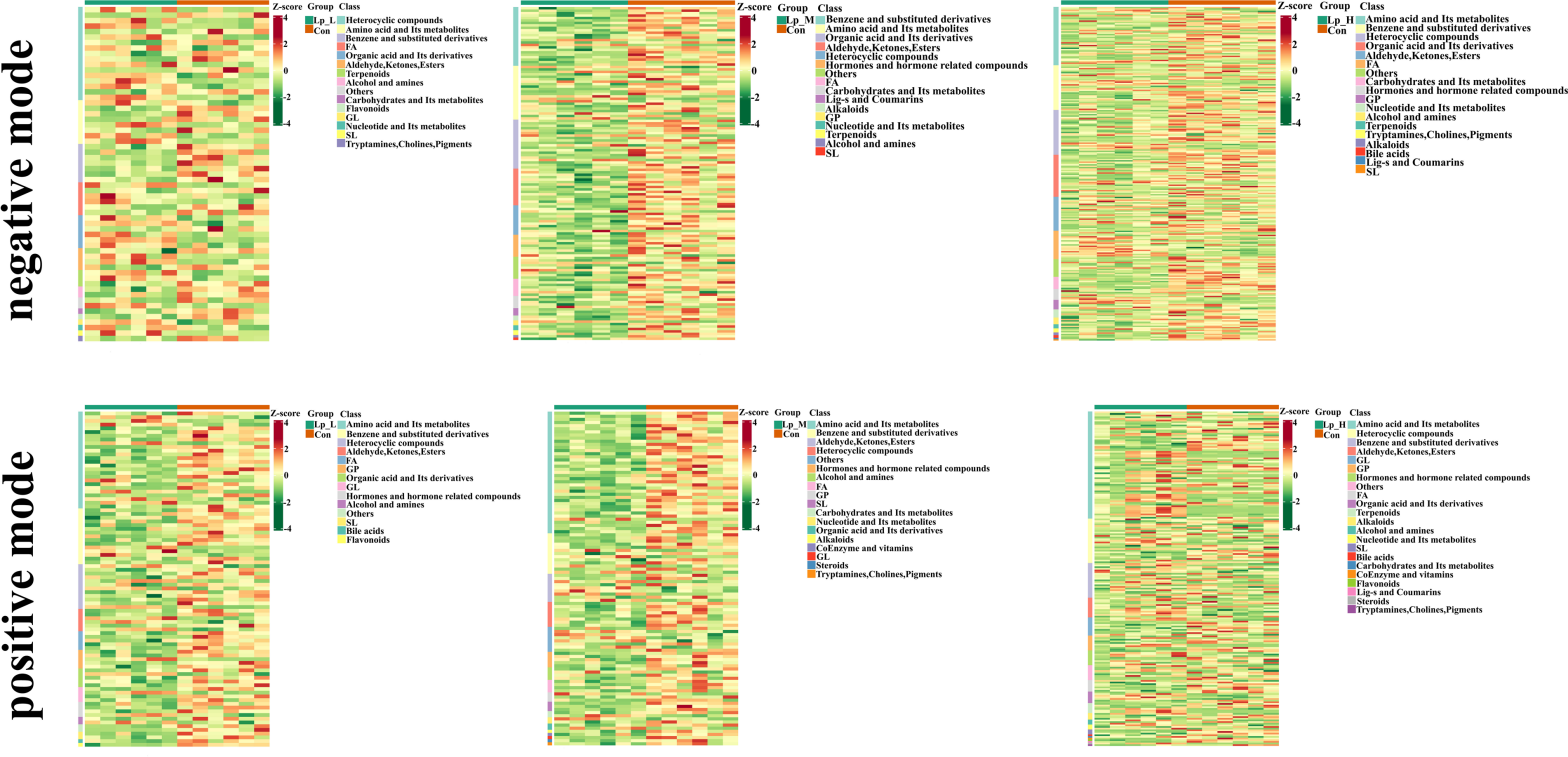
Effects of Lp on the metabolites of the gut microbiota of broilers under positive and negative modes between groups. *n* = 6.

We observed a clear separation from the OPLS-DA score plots between the Lp groups and the Con group (*p* < 0.05) (Figure 3). Based on Figure 4 and Supplementary Tables S5–S7, we found that the Lp_L treatment increased the levels of metabolites in both negative and positive modes, such as PG(16:0/0:0)[U], Tyr-Leu-Ala-Lys, 4-nitrosobiphenyl, and Ser-Trp-Gly (*p* < 0.05). The Lp_L treatment also decreased the levels of metabolites in both negative and positive modes, such as indole-3-acetamide, 6-acetylpicropolin, tazarotene, hydroxysqualene, methyl arachidonate, and morellinol (*p* < 0.05). In addition, the Lp_M treatment induced higher levels of metabolites in both negative and positive modes such as PG(16:0/0:0)[U], Tyr-Leu-Ala-Lys, indoximod, Arg-Val-Ser-Leu-Asp and arachidonoyl Thio-PC (*p* < 0.05). The Lp_M treatment also decreased the levels of metabolites in both negative and positive modes, such as coenzyme Q6, 6-ketomyristic acid (1E, 2S)-2-methylbutanal oxime, and trilinolein (*p* < 0.05). Moreover, the Lp_H treatment increased the levels of metabolites in both negative and positive modes, such as PG(16:0/0:0)[U], furazolidone, paraoxon, gluten exorphin C, and leucomycin A13 (*p* < 0.05). The Lp_H treatment also decreased the levels of metabolites in negative mode such as 6-ketomyristic acid, bosentan, acetyl-Tyr-Val-Ala-Asp-chloromethylketone (*p* < 0.05).

**Figure 5:**
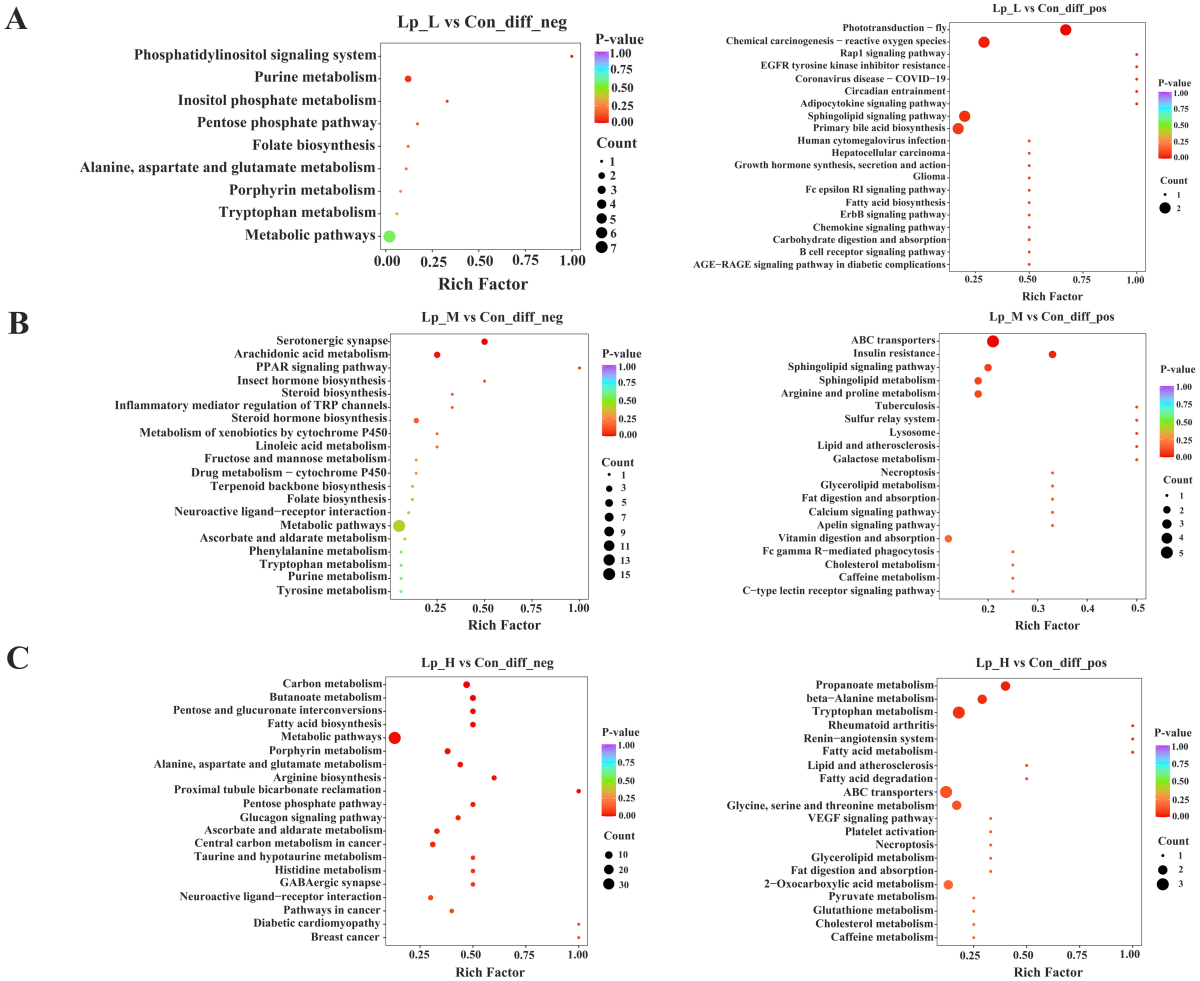
Effects of Lp on the functional prediction of microflora of broilers. *n* = 6.

KEGG analysis revealed that the different metabolites between the Lp_L and Con groups were enriched in pathways including chemical carcinogenesis-reactive oxygen species, sphingolipid signaling pathway, primary bile acid biosynthesis (*p* < 0.05). The different metabolites between the Lp_M and Con groups were enriched in pathways including arachidonic acid metabolism, sphingolipid signaling pathway, arginine and proline metabolism (*p* < 0.05). Moreover, the different metabolites between the Lp_H and Con groups were enriched in pathways including butanoate metabolism, fatty acid metabolism, arginine biosynthesis (*p* < 0.05) (Figure 5).

### 3.7 Correlation analysis

**Figure 6:**
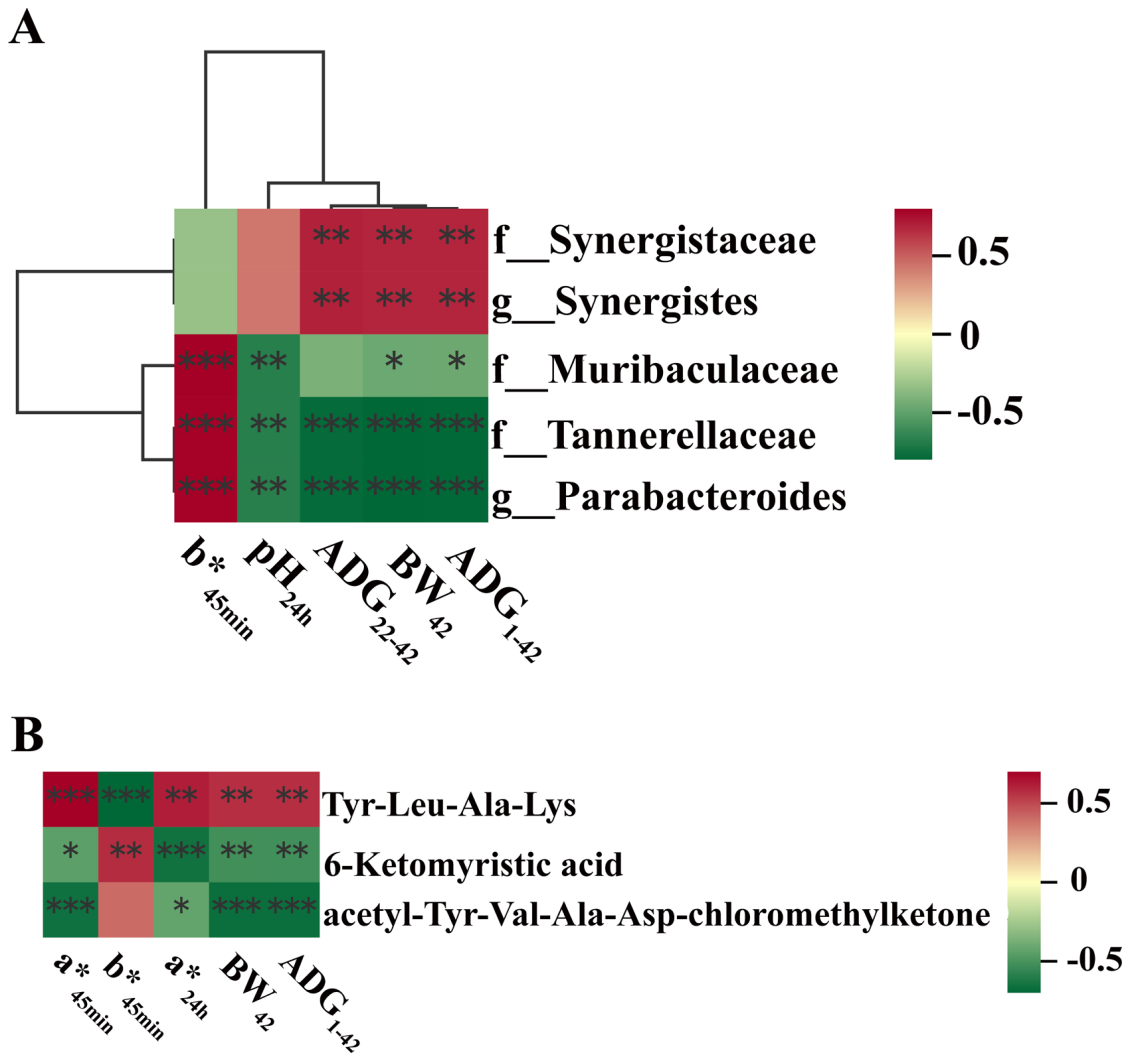
Correlation analyses. **(A)** Correlation analysis between gut microbial genus and growth performance as well as meat quality; **(B)** Correlation analysis between gut microbiota metabolites and growth performance as well as meat quality. Asterisk (*) means FDR-adjusted *p*-values smaller than 0.05. Double asterisks (**) mean FDR-adjusted *p*-values smaller than 0.01. BW, body weight; ADFI, average daily feed intake; ADG, average daily gain; F:G, feed:gain ratio; L*: lightness; a*, redness; b*, yellowness; IMF, intramuscular fat.

Correlation analysis between gut microbial genus and growth performance as well as meat quality was further performed. Results with a correlation coefficient (r) larger than 0.5 or less than −0.5 were selected. Results demonstrated that *Parabacteroides* was negatively correlated with the ADG from day 1 to day 42 (*r* = −0.72; *p* < 0.01), ADG from day 22 to day 42 (*r* = −0.71; *p* < 0.01), BW at day 42 (r = −0.72; *p* < 0.01) and pH_24h_ (*r* = −0.62; *p* < 0.01), and positively correlated with b*_45min_ (*r* = 0.66; *p* < 0.01). Moreover, *Synergistes* was positively correlated with ADG from day 1 to day 42 (*r* = 0.57; *p* < 0.01), ADG from day 22 to day 42 (*r* = 0.57; *p* < 0.01), BW at day 42 (*r* = 0.57; *p* < 0.01) (Figure 6A and Supplementary Table S8).

Correlation analysis between gut microbiota metabolites and growth performance as well as meat quality were also performed. Results with a correlation coefficient (*r*) larger than 0.65 or less than - 0.65 were selected. Results indicated that 6-ketomyristic acid was negatively correlated with a*_24h_ (*r* = −0.65; *p* < 0.01). acetyl-Tyr-Val-Ala-Asp-chloromethylketone was negatively correlated with a*_45min_ (*r* = −0.65; *p* < 0.01), ADG during day 1 to day 42 (*r* = −0.66; *p* < 0.01) and BW at day 42 (*r* = −0.66; *p* < 0.01). Tyr-Leu-Ala-Lys was negatively correlated with b*_45min_ (*r* = −0.69; *p* < 0.01) (Figure 6B and Supplementary Table S9).

## 4 Discussion

Various studies have reported that probiotics can improve the growth performance of broilers. In the present study, the Lp supplementations also increased the BW, ADG and decreased the F:G. Carcass traits can reflect the meat production and economic value of broiler meat. It is shown that the meat production is considered as good if the carcass percentage and eviscerated yield are more than 80 and 60%, respectively (Wang et al., 2024a). Therefore, the increased percentage of eviscerated yield and breast muscle rate suggesting the improvement of meat production by treatments of Lp. Moreover, the Lp_H treatment led to the best growth performance and highest breast muscle rate.

Meat quality is often valued by parameters such as color, pH, water holding capacity, cooking losses, tenderness, or fatty acid content. In the current study, the Lp_H treatment increased the IMF percentage, pH_24h_, a*_45min_, a*_24h_, and decreased the b*_45min_, L*_24h_. The content of IMF is important meat quality characteristic and also related to tenderness, juiciness and flavor (Sun et al., 2023). With the decline of the pH, the meat becomes paler, softer, and higher in drip loss. As for the meat color, the higher the L* value, the paler the meat. A high a* value means an intensive red coloring, while a high b* value indicates the undesirable yellowness. Thus, the above results indicated the improvement of meat quality by the Lp treatments. A range of studies have also described that probiotics affect the physical parameters and chemical parameters of broiler meat. Liu et al. (2022a) reported that *Lactiplantibacillus plantarum* P8 decreased the drip loss and increased the pH in the breast meat of oxidatively-stressed broilers. Zhang et al. (2024) suggested that broilers fed multi-probiotics consisting of *Bacillus subtilis* and *Clostridium butyricum* had a higher pH and water-holding capacity in the breast meat.

The fatty acid plays a role during the regulation for IMF contents and its profiles affect the nutritional and sensory characteristics of meat. As the IMF content was influenced by Lp treatment, we further detected the fatty acid composition in the breast muscle. We found that the fatty acids were mainly C16:0, C18:0, C18:2n6, and C20:4n6, which were consistent with previous study (Xiao et al., 2021). Moreover, increased PUFA/SFA, decreased ∑SFA concentrations and n-6/n-3 ratio were found in the Lp treatments group. Researchers are also interested in the n-6/n-3 ratio in the human diet, as deviations contribute to the pathogenesis of cardiovascular disease, cancer, inflammation, and many autoimmune diseases (Garrison, 2014). Furthermore, the concentrations of C18:3n3 (*α* linolenic acid) was increased by all the Lp treatments, and the content of C20:5n3 (EPA) was also elevated by the Lp_H treatment. C18:3n3 and C20:5n3 are essential fatty acids and C18:3n3 is also the precursor of other PUFA such as EPA, DPA and DHA. A considerable portion of lipid metabolism occurs in the liver. Thus, we further detected the lipid metabolism-related genes in the broiler liver. The gene expressions of *AMPK*, *PPARα*, *CPT-1α, ATGL* were increased, and the expressions of *PPARγ*, *SREBP-1c*, *FAS*, *LPL*, *ACC*, *SCD* were down-regulated by the Lp treatments. *AMPK* mediates lipid metabolism through several lipid metabolism-related transcription factors, such as *PPAR* and *SREBP-1c*. *PPARα* is reported to regulate the expression of lipid oxidation genes, such as *CPT-1α.* Moreover, *PPARγ* induces the expression of *LPL* in adipocytes, thereby promoting the lipid metabolism and lowering blood lipid levels (Vickers et al., 2013). *SREBP-1c* regulates enzymes involved in fatty acid synthesis in the liver, such as *FAS*, *ACC* and *SCD* (Chen et al., 2013). Waters et al. (2009) found a negative relationship between *SCD* gene expression and n-3 PUFA, EPA, DHA and α-linolenic acidin beef cattle, which is in agreement with our present findings where the downregulation of *SCD* occurred in the Lp treatments with the higher n-3 PUFA, EPA, DHA and α-linolenic acid concentrations. Overall, these findings indicated that the Lp supplementations improved the composition of fatty acids in the breast meat of broilers and may potentially improve human health. Taken together, the Lp supplementation improved the fatty acids composition and regulated the lipid metabolism.

The gut microbiota is believed to influence many metabolic processes such as nutrient absorption, host health, and the meat quality. Studies suggested that the regulation of the intestine *Clostridium* increased the composition of n-3 PUFA in the muscle of broilers (Yang et al., 2010), and *Lachnoclostridium* was associated with drip-losing rate, meat fiber diameter, BW, and abdominal fat rate (Lei et al., 2022). In this study, the abundance of Muribaculaceae was decreased in both Lp_L and Lp_M groups. Muribaculaceae is a predominant family of gut Bacteroidales. Similar to our results, Zhou et al. (2023) suggested that feeding fermented herbal residues reduced the drip loss and steaming loss in broiler chicken breasts and also reduced the relative abundance of Muribaculaceae. In the Lp_M and Lp_H groups, the abundance of Tannerellaceae and *Parabacteroides* were also decreased. Tannerellaceae is propionate and butyric-producing bacteria and negatively related to the mRNA levels of *occludin1* in the colon (Zhao et al., 2020). Therefore, the decreased Tannerellaceae may be related to the improvement of intestinal barrier function. *Parabacteroides* represents an opportunistic pathogen in gut, due to its frequent involvement in infectious diseases. It was also linked with the reduction of BW of host animals (Ridaura et al., 2013). Here, the reduced *Parabacteroides* abundance was in accordance with the elevated ADG in Lp_L and Lp_H groups. In addition, Lp_L also induced lower *Merdibacter* and *Colidextribacter* abundances. Although *Merdibacter* was demonstrated to be a beneficial bacterium for gut health in laying hens (Van Hoeck et al., 2021), its roles in regulating meat quality remain unclear. Besides, a serious studies indicated that *Colidextribacter* has beneficial effects on chickens (Liu et al., 2022b), but research also demonstrated that the genus *Colidextribacter* played a role in the increase of cellular oxidative stress capacity (Bhattacharyya et al., 2014). Furthermore, Lp_H also elevated the abundances of Synergistaceae and *Synergistes*, which could ferment amino acids into SCFAs (Yi et al., 2020). A recent study has shown that *Flammulina velutipes* stipe wastes benefits laying hens and also increases Synergistes abundance (Wei et al., 2023). Furthermore, two pathways enriched by the Lp treatments drew our attention: thiamine metabolism and protein export. Thiamine has a well-known significance in the CNS since this vitamin is a regulator of glucose metabolism. Thiamine is involved in functions of multiple enzymes necessary for the metabolism of carbohydrates, fatty acids and amino acids (Rudzki et al., 2021). The enriched protein export pathway indicated that the intake of Lp improved the ability of protein repair of the gut microbiota (Chen et al., 2023b). These results implied that the Lp supplementations improved the structure and function of cecal microbiota of broilers. In addition, we found obvious correlations between the altered gut microbial genus and indices of growth performance and meat quality. For instance, the ADG and BW was negatively correlated with *Parabacteroides* and positively correlated with *Synergistes*. Besides, *Parabacteroide* was also positively correlated with b*_45min_ and negatively correlated with pH_24h_. The above findings imply that the Lp treatment can improve the growth performance and meat quality of broilers by regulating gut microbiota.

The gut microbiota exerts their effects via producing a range of metabolites, which can be used to regulate the glucolipid metabolism and affect the muscle function and meat quality. In the present study, various metabolites were significantly influenced by the supplementation of Lp. With the Lp_L treatment, DL-pantothenic acid level was increased. Report showed that pantothenic acid administration increased the growth performance, slaughter performance, lipid metabolism of geese (Wang et al., 2016). Lp_L also decreased the level of indole-3-acetamide. Dietary tryptophan can be metabolized into indole-3-acetic acid by gut microbiota through indole-3-acetamide pathway under the catalysis of indole-3-acetamide hydrolase (Hubbard et al., 2015). Indole-3-acetic acid alleviated nonalcoholic fatty liver disease in mice via attenuation of hepatic lipogenesis, and oxidative and inflammatory stress (Ji et al., 2019). Therefore, the decreased indole-3-acetamide may imply the production of indole-3-acetic acid via hydrolyzing the indole-3-acetamide. In the Lp_M group, we noticed that the content of C24:1 sphingomyelin was decreased. C24:1 sphingomyelin has a solubilizing effect on liquid-ordered domains, with a capacity to accommodate other lipids in a single phase in model membranes even at the nanometric scale (Maté et al., 2014). Additionally, the level of saikosaponin A was increased in the Lp_H group. Saikosaponin A can inhibit the inflammation (Lu et al., 2012) and suppress adipogenesis in 3 T3-LI adipocytes (Lim et al., 2021). Moreover, the Lp_L treatment enriched the pathways such as primary bile acid biosynthesis. Bile acids, primarily synthesized in the liver from cholesterol (Hofmann and Hagey, 2008), can solubilize dietary fat and promote fat absorption and glucose homeostasis when secreted into the intestine (Zhang et al., 2022). Studies showed that supplementation of diets with 60 and 80 mg/kg of bile acid derived from swine can effectively improve growth performance and carcass traits of broilers (Lai et al., 2018). The Lp_L and Lp_H treatments enriched the pathways related to amino acids and lipid metabolism, such as the arginine and proline metabolism, fatty acid metabolism and arginine biosynthesis pathways, implying that these altered metabolites were able to metabolize large organic molecules to produce amino acids and fatty acids. Additionally, obvious correlations were found between the altered gut microbial metabolites and indices of growth performance and meat quality. We found that the 6-ketomyristic acid was negatively correlated with a*_24h_. Acetyl-Tyr-Val-Ala-Asp-chloromethylketone was negatively correlated with a*_45min_, ADG and BW. Tyr-Leu-Ala-Lys was negatively correlated with b*_45min_. Taken together, the Lp treatment improved the growth performance and meat quality of broilers by regulating gut microbiota metabolites.

## 5 Conclusion

In conclusion, our data show that the Lp supplementation improved the growth performance, meat quality, carcass traits, fatty acids composition, lipid metabolism-related gene expressions, gut microbiota and metabolites in broilers, and the high level Lp had a more significant effect. Moreover, based on the correlation analysis, we found that Lp may increase the growth performance and meat quality by regulating the gut microbiota (*Synergistes*, etc.) and its metabolites (6-ketomyristic acid, etc.). However, the underling mechanisms of these gut microbiota and metabolites need further studies.

## Data Availability

The datasets presented in this study can be found in online repositories. The names of the repository/repositories and accession number(s) can be found in the article/Supplementary material.
