# Supplementary material for: Lactiplantibacillus plantarum improves the growth performance and meat quality of broilers by regulating the cecal microbiota and metabolites
Source: Front Microbiol. 2025 Jan 28;16:1519552. doi: 10.3389/fmicb.2025.1519552 (PMC11811115; doi:10.3389/fmicb.2025.1519552)
Supplement: Supplementary file 1 [file Data_Sheet_1.docx]

Supplementary Material

**Supplementary Table S1** Gene primers

| **Gene** | **Forward Primer sequence（5'-3')** | **Reverse Primer sequence（5'-3')** |
| --- | --- | --- |
| *β-actin* | TGATATTGCTGCGCTCGTTG | AACCATCACACCCTGATGTCTG |
| *AMPK* | GATATTTGGAGCAGTGGGGTTA | GGAAACAAGTATTTGGGAAGGT |
| *CPT-1α* | GACGGACACTGCAAAGGAGA | GGCATCAGGGCTGGTTTTTG |
| *PPARα* | TAACGGAGTTCCAATCGC | AACCCTTACAACCTTCACAA |
| *PPARγ* | TGACAGCGCCAGAGATTACA | CATCCATCGCAGACAGATCCA |
| *SREBP-1c* | GTCGGCGATCCTGAGGAA | CTCTTCTGCACGGCCATCTT |
| *FAS* | GCGGTAGCGAGACGGG | ATAACTAACACAATGATGAGGAGCA |
| *SCD* | CCAGAAGCTGGACCTGAGTG | GGGCTTGTTAGTATCTCCGCT |
| *LPL* | TGGACATTGGTGACCTGCTTATGC | TCGCCTGACTTCACTCTGACTCTC |
| *ACC* | TCCCACCCAAACAGAATGTCC | AGAGACCATCCCTCCCATCC |
| *ATGL* | GTTCATCTCTTCCTCCGACCC | AAGCGGAGTCCAAAGGGAAC |

**Supplementary Table S2** Significantly influenced bacteria abundance between Lp_L and Con groups

| **Bacteria** | **Lp_L** | **Con** | **up/down** | **log2FC** | ***p*-value** |
| --- | --- | --- | --- | --- | --- |
| f__Muribaculaceae | 0.000 010 | 0.000 076 | down | -2.91 | 0.016 |
| f__Tannerellaceae | 0.011 | 0.051 | down | -2.14 | 0.030 |
| g__*Merdibacter* | 0.000 10 | 0.000 54 | down | -2.39 | 0.012 |
| g__*Colidextribacter* | 0.004 7 | 0.009 6 | down | -1.02 | 0.016 |
| g__*Parabacteroides* | 0.011 | 0.050 | down | -2.15 | 0.030 |

FC, fold change, n = 6.

**Supplementary Table S3** Significantly influenced bacteria abundance between Lp_M and Con groups

| **Bacteria** | **Lp_M** | **Con** | **up/down** | **log2FC** | **p-value** |
| --- | --- | --- | --- | --- | --- |
| f__Muribaculaceae | 0.000 005 1 | 0.000 076 | down | -3.91 | 0.012 |

FC, fold change, n = 6.

**Supplementary Table S4** Significantly influenced bacteria abundance between Lp_H and Con groups

| **Bacteria** | **Lp_H** | **Con** | **up/down** | **log2FC** | **p-value** |
| --- | --- | --- | --- | --- | --- |
| f__Synergistaceae | 0.017 0 | 0.003 9 | up | 2.13 | 0.017 |
| f__Tannerellaceae | 0.008 4 | 0.050 5 | down | -2.59 | 0.023 |
| g__*Synergistes* | 0.016 9 | 0.003 8 | up | 2.14 | 0.016 |
| g__*Parabacteroides* | 0.008 3 | 0.050 5 | down | -2.60 | 0.024 |

FC, fold change, n = 6.

**Supplementary Table S5** Significantly influenced cecal bacteria metabolite between Lp_L and Con groups (Top 10)

| **Negative mode** | **Lp_L** | **Con** | **up/down** | **log2FC** | ***p*-value** |
| --- | --- | --- | --- | --- | --- |
| Isolimonic acid 16->17-lactone | 35 312.35 | 6 685.11 | up | 2.40 | 0.021 |
| PG(16:0/0:0)[U] | 204 775.40 | 39 922.82 | up | 2.36 | 0.009 |
| Tyr-Leu-Ala-Lys | 83 796.30 | 19 846.70 | up | 2.08 | 0.024 |
| N-Palmitoylsphingomyelin | 3 991.86 | 1 098.13 | up | 1.86 | 0.037 |
| DL-Pantothenic acid | 74 039.76 | 21 019.86 | up | 1.82 | <0.001 |
| Sarcodon scabrosus Depsipeptide | 226 909.50 | 65 250.54 | up | 1.80 | 0.023 |
| Indole-3-acetamide | 1 492.56 | 6 622.90 | down | -2.15 | 0.007 |
| 6-Acetylpicropolin | 65 540.86 | 207 798.04 | down | -1.66 | 0.043 |
| Tazarotene | 455.56 | 1 420.62 | down | -1.64 | 0.024 |
| alpha-Ketoglutaramate | 2 183.21 | 6 628.19 | down | -1.60 | 0.047 |
| **Positive mode** | **Lp_L** | **Con** | **up/down** | **log2FC** | ***p*-value** |
| 1,2-Dioleoyl-sn-Glycero-3-Phosphocholine | 195 309.12 | 18 306.77 | up | 3.42 | 0.024 |
| 4-Nitrosobiphenyl | 180 484.56 | 31 705.92 | up | 2.51 | 0.010 |
| Ser-Trp-Gly | 40 682.82 | 11 731.45 | up | 1.79 | 0.045 |
| Ginsenoyne M | 1 644 944.02 | 480 465.95 | up | 1.78 | 0.015 |
| Hydroxysqualene | 2 326.56 | 28 041.61 | down | -3.59 | 0.032 |
| Methyl arachidonate | 9 258.58 | 49 863.76 | down | -2.43 | 0.037 |
| Morellinol | 23 249.58 | 102 654.63 | down | -2.14 | 0.041 |
| Ile-Thr-His-Asp | 29 347.23 | 122 362.82 | down | -2.06 | 0.020 |
| Thr-Arg-Gly-Glu-Val | 2 203.01 | 8 127.34 | down | -1.88 | 0.034 |
| 7alpha,24-Dihydroxycholest-4-en-3-one | 43 855.67 | 153 359.74 | down | -1.81 | 0.017 |

FC, fold change, n = 6.

**Supplementary Table S6** Significantly influenced cecal bacteria metabolite between Lp_M and Con groups (Top 10)

| **Negative mode** | **Lp_M** | **Con** | **up/down** | **log2FC** | ***p*-value** |
| --- | --- | --- | --- | --- | --- |
| PG(16:0/0:0)[U] | 392 858.59 | 39 922.82 | up | 3.30 | 0.027 |
| Tyr-Leu-Ala-Lys | 122 910.99 | 19 846.70 | up | 2.63 | 0.005 |
| Sarcodon scabrosus depsipeptide | 348 295.06 | 65 250.54 | up | 2.42 | 0.001 |
| Indoximod | 136 985.67 | 36 496.25 | up | 1.91 | 0.007 |
| 3-Nonanon-1-yl acetate | 3 239.70 | 15 802.71 | down | -2.29 | 0.020 |
| Coenzyme Q6 | 18 683.15 | 73 681.38 | down | -1.98 | 0.045 |
| 6-Ketomyristic acid | 2 669.61 | 10 351.61 | down | -1.96 | 0.040 |
| Vanillic acid 4-sulfate | 4 431.06 | 16 186.69 | down | -1.87 | 0.026 |
| 2-Methyl-4-pentenoic acid | 5 867.19 | 21 376.54 | down | -1.87 | 0.019 |
| Ile-Glu-Val-Asp-Leu | 29 177.96 | 100 897.16 | down | -1.79 | 0.015 |
| **Positive mode** | **Lp_M** | **Con** | **up/down** | **log2FC** | ***p*-value** |
| Arg-Val-Ser-Leu-Asp | 976.21 | 70.00 | up | 3.80 | 0.036 |
| Arachidonoyl Thio-PC | 421 914.92 | 128 137.82 | up | 1.72 | 0.001 |
| (1E,2S)-2-methylbutanal oxime | 1 544.40 | 9 414.51 | down | -2.61 | 0.044 |
| Asn-Lys-Ala-Val-Gly | 13 576.05 | 72 155.93 | down | -2.41 | 0.043 |
| Trilinolein | 1 221.55 | 5 381.19 | down | -2.14 | 0.020 |
| Leu-Ser | 5 350.08 | 23 383.35 | down | -2.13 | 0.035 |
| C24:1 Sphingomyelin | 6 170.39 | 25 041.69 | down | -2.02 | <0.001 |
| Ser-Ser-Gly | 16 203.45 | 62 190.97 | down | -1.94 | 0.042 |
| PC(18:3(6Z,9Z,12Z)/P-18:1(11Z)) | 19 005.33 | 69 687.03 | down | -1.87 | 0.020 |
| Met-Gly-Ile | 28 368.44 | 91 617.58 | down | -1.69 | 0.007 |

FC, fold change, n = 6.

**Supplementary Table S7** Significantly influenced cecal bacteria metabolite between Lp_H and Con groups (Top 10)

| **Negative mode** | **Lp_H** | **Con** | **up/down** | **log2FC** | ***p*-value** |
| --- | --- | --- | --- | --- | --- |
| PG(16:0/0:0)[U] | 666 772.56 | 39 922.82 | up | 4.06 | 0.004 |
| 7-Hydroxy-2,5-dimethyl-4H-1-benzopyran-4-one | 73 204.73 | 8 979.14 | up | 3.03 | 0.001 |
| Furazolidone | 118 650.32 | 15 971.17 | up | 2.89 | 0.001 |
| Paraoxon | 10 009.58 | 1 350.86 | up | 2.89 | 0.003 |
| Tyr-Leu-Ala-Lys | 139 484.01 | 19 846.70 | up | 2.81 | <0.001 |
| 6-Ketomyristic acid | 999.93 | 10 351.61 | down | -3.37 | 0.020 |
| 7-[(2R)-3-hydroxy-2-(3-hydroxy-5-methylnon-1-enyl)-5-oxocyclopentyl]-3-methoxyheptanoic acid | 1 245.93 | 12 274.47 | down | -3.30 | 0.048 |
| Bosentan | 287.05 | 2 417.70 | down | -3.07 | 0.002 |
| acetyl-Tyr-Val-Ala-Asp-chloromethylketone | 2 853.05 | 23 704.59 | down | -3.05 | 0.006 |
| [2,4-dihydroxy-3-[(E)-3-methylbut-1-enyl]phenyl]-[6-(2,4-dihydroxyphenyl)-2-(2,6-dihydroxyphenyl)-4-methylcyclohex-3-en-1-yl]methanone | 839.47 | 6 419.10 | down | -2.93 | 0.005 |
| **Positive mode** | **Lp_H** | **Con** | **up/down** | **log2FC** | ***p*-value** |
| Gluten Exorphin C | 36 716.33 | 81.95 | up | 8.81 | 0.010 |
| (3b,22a)-12-Oleanene-3,22,24,29-tetrol 3-[arabinosyl-(1->3)-arabinoside] | 20 461.84 | 46.30 | up | 8.79 | 0.012 |
| Leucomycin A13 | 16 114.92 | 43.39 | up | 8.54 | 0.013 |
| Arg-Val-Ser-Leu-Asp | 16 126.07 | 70.00 | up | 7.85 | 0.010 |
| Gly-Leu-Arg-Asn-Gln | 10 029.65 | 116.62 | up | 6.43 | 0.017 |
| Asp-Gly-Lys-Ile-Leu | 12 305.23 | 145.64 | up | 6.40 | 0.012 |
| Saikosaponin A | 10 005.66 | 135.85 | up | 6.20 | 0.014 |
| Arg-Glu-Lys-Asp-Lys | 21 441.95 | 356.77 | up | 5.91 | 0.013 |
| Hexadecanoyl-2-octadecanoyl-sn-glycero-3-phosphocholine | 14 466.69 | 248.91 | up | 5.86 | 0.033 |
| Tyr-His-Arg-Arg | 31 715.67 | 595.94 | up | 5.73 | 0.009 |

FC, fold change, n = 6.

**Supplementary Table S8** Correlation analysis between gut microbial genus and growth performance as well as meat quality indices

| **Gut microbial genus** | **Growth performance** | **r-value** | ***p*-value** |
| --- | --- | --- | --- |
| g__*Synergistes* | ADG_1-42_ | 0.57 | 0.001 |
| g__*Synergistes* | BW_42_ | 0.57 | 0.001 |
| g__*Synergistes* | ADG_22-42_ | 0.57 | <0.001 |
| g__*Parabacteroides* | pH_24h_ | -0.62 | 0.042 |
| g__*Parabacteroides* | b*_45min_ | 0.66 | 0.002 |
| g__*Parabacteroides* | ADG_22-42_ | -0.71 | 0.002 |
| g__*Parabacteroides* | ADG_1-42_ | -0.72 | 0.001 |
| g__*Parabacteroides* | BW_42_ | -0.72 | 0.001 |

**Supplementary Table S9** Correlation analysis between gut microbial metabolites and growth performance as well as meat quality indices

| **Gut microbial metabolites** | **Growth performance** | **r-value** | ***p*-value** |
| --- | --- | --- | --- |
| 6-Ketomyristic acid | a*_24h_ | -0.65 | 0.001 |
| acetyl-Tyr-Val-Ala-Asp-chloromethylketone | a*_45min_ | -0.65 | 0.001 |
| acetyl-Tyr-Val-Ala-Asp-chloromethylketone | ADG_1-42_ | -0.66 | <0.001 |
| acetyl-Tyr-Val-Ala-Asp-chloromethylketone | BW_42_ | -0.66 | <0.001 |
| Tyr-Leu-Ala-Lys | b*_45min_ | -0.69 | <0.001 |
